# The Way Day Breaks

**DOI:** 10.1007/s40596-021-01410-5

**Published:** 2021-02-24

**Authors:** Emily Duggan

**Affiliations:** Boston, MA USA

“This is the way day breaks in Bowditch Hall at Mclean’s” — Robert Lowell, “Waking in the Blue”

i.

Waking in the black that comes for the living

only before dawn, we’re wrung from our beds

like damp hand towels: used; leaking blood

(some of us). They take our weights & vitals

in vials, one by one; the rest restlessly await

our next & nightly judgment, draped across

the vile mauve milieu couches like what

the purple sea coughs up, looking at

(without seeing) the five A.M. news:

eating disorders, the newscaster claims, happen most often to

*good people*. This *roomful of good people* (what better

collective noun for us than this?) giggles,

or nearly cries. Leslie Nielsen dies quietly

& surely in the night. & a boy my age, already dead, falls

from the airplane’s wheel well in real time

into a nearby city, where a fellow runner finds

his body.

ii.

*Perfection* has always been the story

my body has wanted to tell —

& for everyone who has ached

to understand how *perfection* can align

so intuitively with *self-destruction*,

there is someone who has told me

they’d kill

for my control.

iii.

Well –

I tried.

iv.

Waking in the white that comes for the dead

only before waking, everything is still,

the same, another morning at Mclean’s,

the steady drag of traffic on the pre-light highways,

the blackened cars moving behind their headlights

like the baggage ghosts carry past,

& all I can see

is the boy from the plane:

my age. The anguish

on that runner’s face:

their hell. & I,

who half my life have sleepwalked

into the sky, wake up halfway to the ground, eyes

ripped wide:

v.

*this is not the story I want to live to tell*.

vi.

I am sick to death of wanting to die. I want all that time

back. I want to burst back in time, cradle

the soft, fearful corpse-in-waiting I have been

until they feel safe inside those rabbit bones

– home. & alive,

for the first time.

But I don’t want to die –

not today.

Tomorrow – yes – FINE –

vii.

but *today*

viii.

I am standing up & climbing back

inside the airplane. I’m taking us in

for a safe landing. I want all the pieces

of me together at the end,

so my discoverers can say,

*AHA! So these are the hands with which*

*they dismantled the murder machine*

*of their mind. These are the legs*

*that kicked back. & these –*

*THESE –*

*etched around their eyes*

*like the rings of a tree –*

*these are the years they lived after*

*in whatever approximates peace:*

*that which runs parallel to perfection,*

*equivalent but untouching.*

**Poet’s statement:**

Written in the first person to illuminate the speaker’s authority on their experience of the poem’s content, I hope this piece can prove valuable in providing one perspective from a person in treatment on the potentially transformative nature of psychiatric care.

